# Essential oils affect populations of some rumen bacteria *in vitro* as revealed by microarray (RumenBactArray) analysis

**DOI:** 10.3389/fmicb.2015.00297

**Published:** 2015-04-10

**Authors:** Amlan K. Patra, Zhongtang Yu

**Affiliations:** ^1^Department of Animal Sciences, The Ohio State UniversityColumbus, OH, USA; ^2^Department of Animal Nutrition, West Bengal University of Animal and Fishery SciencesKolkata, India

**Keywords:** essential oil, bacterial composition, microarray, rumen, RumenBactArray

## Abstract

In a previous study origanum oil (ORO), garlic oil (GAO), and peppermint oil (PEO) were shown to effectively lower methane production, decrease abundance of methanogens, and change abundances of several bacterial populations important to feed digestion *in vitro*. In this study, the impact of these essential oils (EOs, at 0.50 g/L) on the rumen bacterial community composition and population was further examined using the recently developed RumenBactArray. Species richness (expressed as number of operational taxonomic units, OTUs) in the phylum *Firmicutes*, especially those in the class *Clostridia*, was decreased by ORO and GAO, but increased by PEO, while that in the phylum *Bacteroidetes* was increased by ORO and PEO. Species richness in the genus *Butyrivibrio* was lowered by all the EOs. Increases of *Bacteroidetes* OTUs mainly resulted from increases of *Prevotella* OTUs. Overall, 67 individual OTUs showed significant differences (*P* ≤ 0.05) in relative abundance across the EO treatments. The predominant OTUs affected by EOs were diverse, including those related to *Syntrophococcus sucromutans*, *Succiniclasticum ruminis*, and *Lachnobacterium bovis*, and those classified to *Prevotella*, *Clostridium*, *Roseburia*, *Pseudobutyrivibrio*, *Lachnospiraceae*, *Ruminococcaceae*, *Prevotellaceae, Bacteroidales*, and *Clostridiales*. In total, 60 OTUs were found significantly (*P* ≤ 0.05) correlated with feed degradability, ammonia concentration, and molar percentage of volatile fatty acids. Taken together, this study demonstrated extensive impact of EOs on rumen bacterial communities in an EO type-dependent manner, especially those in the predominant families *Prevotellaceae*, *Lachnospiraceae*, and *Ruminococcaceae*. The information from this study may aid in understanding the effect of EOs on feed digestion and fermentation by rumen bacteria.

## Introduction

In recent years, a variety of plant bioactive compounds, including saponins, essential oils (EOs), tannins, and flavonoids have been evaluated for their ability to modulate rumen microbial fermentation processes to improve feed utilization efficiency while decreasing methane emission and nitrogen excretion (Patra and Saxena, 2009, 2010). EOs have received more research interest than other types of plant bioactive compounds because they can effectively improve several important aspects of microbial metabolism in the rumen. For example, EOs can slow down degradation of starch and protein degradation, thereby reducing the risk of rumen acidosis in cattle fed high concentrate diets and decreasing intra-ruminal nitrogen turnover and nitrogen excretion, respectively, while inhibiting methanogenesis (McIntosh et al., 2003; Calsamiglia et al., 2007; Patra, 2011). Several studies also showed that supplementation of EOs to dairy cows resulted in increased milk yield and feed efficiency (Kung et al., 2008; Tassoul and Shaver, 2009; Giannenas et al., 2011). Although EOs have shown promise to inhibit the methanogenic archaea and methane production in the rumen (Patra and Saxena, 2010), adverse effects on fiber digestion and fermentation have also been reported, with the magnitude of these adverse effects varying considerably depending upon the type and dose of EO and diet composition (Calsamiglia et al., 2007; Macheboeuf et al., 2008). Determination of the effect of EOs on rumen bacterial communities is essential to understanding how EOs influence feed digestion and fermentation.

Phylogenetic microarrays enable simultaneous detection and semi-quantitation of thousands of different members of a microbiome (Rajilic-Stojanovic et al., 2009; Schatz et al., 2010). They have been used in investigations of bacteria in various environments, such as soil, human gut, human feces, sludges, and lakes (Small et al., 2001; Adamczyk et al., 2003; Castiglioni et al., 2004; Palmer et al., 2006; Rajilic-Stojanovic et al., 2009; Kang et al., 2010). A phylochip specifically for comprehensive analysis of rumen bacterial communities was recently developed based on 16S rRNA gene sequence with operational taxonomic units (OTUs) calculated at 97% sequence similarity (Kim et al., 2014). This phylochip, referred to as RumenBactArray, has more than 1600 OTU-specific probes that allow detection and semi-quantification of rumen bacteria. The RumenBactArray detects as few as 10^6^ copies of a target and has a linear detection range of >4 orders of magnitude. The objectives of the present study were to assess the effect of three different EOs, which were shown to inhibit methanogenesis and modify rumen fermentation characteristics and select microbial populations and community (Patra and Yu, 2012), on the bacterial communities using the new RumenBactArray, and to identify associations between microbial populations, and digestion and fermentation variables. Broad effects were revealed and different sets of bacterial groups were affected differently by origanum oil (ORO), garlic oil (GAO), and peppermint oil (PEO). The results may help better understand the effect of these EOs on feed digestion and fermentation in the rumen.

## Materials and methods

### Source of DNA samples

The DNA samples analyzed in the present study had been analyzed previously using DGGE and qPCR (Patra and Yu, 2012). In that study, clove oil (CLO), eucalyptus oil (EUO), GAO, ORO, and PEO were evaluated *in vitro* at different doses (0.25, 0.50, and 1.0 g/L) for their effects on methane production, feed digestion, and fermentation. Their effects on communities of bacteria and of archaea were examined using DGGE, while changes in abundances of total bacteria, total archaea, total protozoa, and select cellulolytic bacteria (including *Fibrobacter succinogenes, Ruminococcus flavefaciens*, and *Ruminococcus albus*) were determined using specific qPCR. Different EOs were found to have different effect on most of the measurements in a dose dependent manner (Patra and Yu, 2012). In the present study, the effect of three of the EOs was further evaluated using RumenBactArray. These three EOs included GAO, ORO, and PEO. These three EOs were chosen because their principal bioactive components represent different chemical structures and stereochemistry: GAO contains alliin and allicin (organosulphur compounds); ORO contains thymol (monoterpinoid monoclyclic phenol); and PEO contains menthol (monoterpinoid monoclyclic non-phenol). Only the *in vitro* cultures (three replicates) that received 0.50 g/L each EO were used in the present study. This dose generally resulted in mild negative effects on digestion and rumen fermentation compared with high concentration (1 g/L). The control culture (three replicates) that did not receive EO was included in parallel. The detailed procedures of the *in vitro* experiment, sampling, and DNA extraction are available in the recent paper by Patra and Yu (2012).

### Sample preparation, labeling, and microarray hybridization

Samples were prepared, labeled, and then subjected to microarray hybridization as described previously (Kim et al., 2014). Briefly, nearly full-length 16S rRNA genes were amplified from each metagenomic DNA sample using the universal primer set 27F (5′-AGA GTT TGA TCM TGG CTC AG-3′) and T7/1492R (5′-TCT AAT ACG ACT CAC TAT AGG GGG YTA CCT TGT TAC GAC TT-3′) as described previously. The amplicons were purified using a PCR purification Kit (Qiagen, Valencia, CA, USA) and then used in preparation of complementary RNA (cRNA) using a MEGAScript T7 *in vitro* transcription kit (Ambion, Austin, TX, USA). Following purification of the cRNA using a MEGAclear kit (Ambion, Austin, TX, USA), the cRNA was labeled with Cy5 at 37°C for 1 h using a Label IT®μ ArrayCy3/Cy5 Labeling kit (Mirus, Madison, WI, USA). The labeled cRNA was again purified to remove the free Cy5 dye using a MEGAclear kit. The labeled cRNA was quantified using the NanoDropt™ 1000 spectrophotometer, and then stored at −80°C until microarray hybridization.

Microarray hybridization was performed using Agilent Technologies' Hybridization gasket slides as described previously (Kim et al., 2014). Briefly, the hybridization solution containing 6× SSPE, 0.01% Tween-20, 0.01 mg/ml acetylated bovine serum albumin (BSA), 10% formamide, and 150 ng labeled cRNA was incubated at 65°C for 5 min and then placed on ice for 5 min. The Agilent hybridization cassette, the Agilent gasket slide and the microarray slide were preheated at 65°C while the hybridization solution was prepared. The hybridization solution was added to the center of the Agilent gasket slide, and then the RumenBactArray slide was placed over the gasket slide. The assembled cassette was placed in a HB-1000 hybridization oven (UVP, LLC) preset at 45°C to allow hybridization for 18 h with rotation set at 10 rpm. After hybridization, the microarray slides were bathed in 1× SSPE buffer (45°C) for 3 min twice and then in 0.25× SSPE for 30 s once prior to drying by centrifugation for 2 min at 400 g at room temperature.

### Signal detection and data analysis

The microarray slides were scanned and the fluorescent images of the hybridized microarrays were analyzed as previously described (Kim et al., 2014). Briefly, the hybridization images were captured with a GenePix 4000B Scanner (Axon Instruments, Union City, CA). The Cy5 fluorescence signal at each probe spot was measured using the GenePix®Pro 6.0 program (Axon Instruments). Probe spots on the scanned images were recognized by manually superimposing the gene allocation list (GAL) file provided by the manufacturer, which carries the annotation information of each spot on the microarray, over scanned images. Poor-quality probe spots that had a signal intensity lower than the background threshold, irregular size, or overlap with an adjacent spot were excluded from further analysis. Images were also inspected manually, and probe spots in low-quality areas of the microarray were also flagged and excluded from further analysis. The local median background signal intensity was subtracted from the median hybridization signal intensity of each separate probe spot. After background subtraction, normalization was performed based on the signal intensity of internal control probes targeting the bovine mitochondrial rRNA gene. Relative abundance of each OTU was calculated as its probe signal intensity percentage of total bacterial probe signal intensity. To assess if relative abundance of each bacterial OTU differed significantly (*P* ≤ 0.05) or tended (0.05 <P ≤ 0.10) to differ between control and the EO treatments, One-Way ANOVA was performed. When *P* ≤ 0.05, Tukey's test was employed to determine significant differences among the treatments. Both raw and normalized data are available in NCBI GEO under accession number GSE62624. Principal component analysis (PCA) using the MeV program (Saeed et al., 2006) was performed to compare the bacterial communities among the samples. The PCA scores on the first three principal components were further analyzed by multivariate analysis of variance (MANOVA) to test for differences in community composition among the treatments using SAS (2001).

Pearson correlation coefficients were calculated using SAS (2001) to examine correlation between relative abundances of each bacterial OTU and each of the fermentation data, including dry matter degradability (DMD), concentrations of ammonia, and molar percentages of acetate, propionate and butyrate, which were obtained in the previous study (Patra and Yu, 2012). Significant correlation was considered at *P* ≤ 0.05.

## Results

### Effects of EOs on richness and distribution of ruminal bacteria

**Figure 1:**
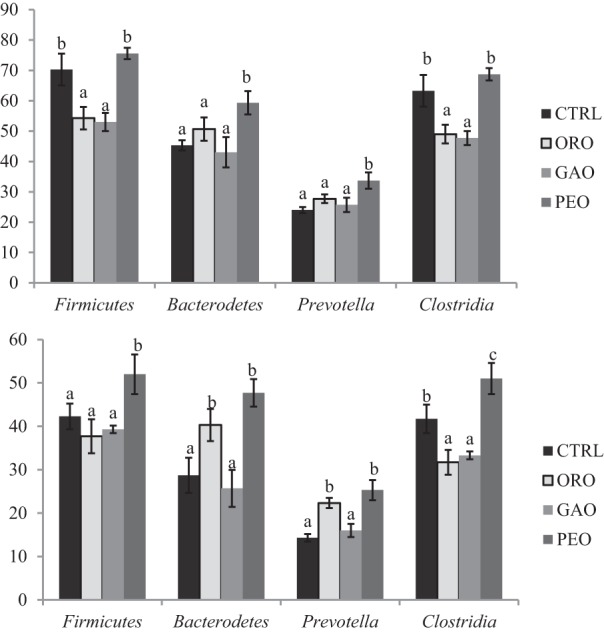
**Average number of major bacterial OTUs (relative abundance of ≥0.5 or ≥1%) identified in *Firmicutes, Bacteroidetes, Clostridia*, and *Prevotella***. CTRL, control; ORO, origanum oil; GAO, garlic oil; PEO, peppermint oil; Different letters (a–c) above the bars indicate significant (*P* ≤ 0.05) differences among the treatments (three replicates per treatment).

The species richness, expressed as numbers of OTUs detected, was affected by all the EO treatments (Table 1). Overall, 228 OTUs with relative abundances greater than 0.5% were identified within different phyla among all the treatments. Of the OTUs with a relative abundance ≥0.5% (of total bacterial probe signal intensity), the number of OTUs in the phylum *Firmicutes* was considerably lower, especially in the class *Clostridia*, in the cultures that received ORO or GAO than in the control (Figure 1). However, the number of OTUs with a relative abundance ≥1.0% was increased by PEO compared with the control, while the number of OTUs with a relative abundance ≥0.5% was similar between the control and the PEO cultures. Compared to the control, all the EO treatments decreased the number of OTUs in the genus *Butyrivibrio*. The numbers of OTUs with a relative abundance ≥0.5% and unclassified within *Ruminococcaceae* were decreased by GAO and ORO. The numbers of OTUs with a relative abundance ≥1% in the phylum *Bacteroidetes* were increased by ORO and PEO, but were not affected by GAO; whereas the numbers of OTUs with a relative abundance ≥0.5% in this phylum was greater for PEO, but not for ORO or GAO, than for the control. The increases of OTU richness in *Bacteroidetes* mainly resulted from increases of *Prevotella* OTUs. No significant changes were observed in the number of OTUs classified to other phyla, families, or genera for any of the EO treatments.

**Table 1:** **Effects of essential oils on richness and distribution of major ruminal bacterial taxa**.

	**Relative abundance ≥ 1%**				**Relative abundance ≥ 0.50%**			
	**C**	**ORO**	**GAO**	**PEO**	**C**	**ORO**	**GAO**	**PEO**
**Phylum *Firmicutes***	**46**	**37**	**38**	**56**	**77**	**62**	**57**	**80**
**Class *Bacilli***	**2**	**3**	**3**	**3**	**3**	**3**	**4**	**5**
*Bacillus*	1	1	1	1	1	1	1	1
*Carnobacterium*	0	0	0	0	0	0	0	1
*Lactobacillus*	0	0	1	0	0	0	1	0
*Streptococcus*	1	2	1	2	2	2	2	2
*Pasteuriaceae incertae sedis*	0	0	0	0	0	0	0	1
**Class *Clostridia***	**43**	**33**	**34**	**51**	**70**	**55**	**51**	**73**
Family *Veillonellaceae*								
*Megasphaera*	0	0	1	0	0	0	1	0
*Succiniclasticum*	3	1	2	2	3	2	2	3
*Mitsuokella*	0	1	0	1	2	3	3	3
*Dialister*	1	0	1	0	1	1	1	1
U_*Veillonellaceae*	1	1	1	1	2	2	1	1
Family *Incertae sedis XIII*								
*Anaerovorax*	1	0	0	0	1	0	0	2
*Mogibacterium*	1	0	0	0	1	0	1	0
U_*Incertae sedis XIII*	0	1	0	0	1	1	1	1
Family *Ruminococcaceae*								
*Acetivibrio*	0	0	0	0	1	0	0	0
*Acetanaerobacterium*	1	0	0	0	1	0	0	0
*Papillibacter*	1	1	1	2	2	2	1	2
*Ruminococcus*	2	2	1	3	3	3	3	3
*Sporobacter*	1	1	1	1	1	2	1	2
U_*Ruminococcaceae*	6	6	5	8	12	7	6	10
Family *Clostridiaceae*								
*Clostridium*	0	0	1	1	0	0	2	2
Family U*_Peptococcaceae*	0	0	0	1	0	0	0	1
Family *Incertae sedis XI*								
*Sedimentibacter*	1	0	0	0	1	0	0	0
Family *Peptostreptococcaceae_IS*	0	0	0	0	0	0	0	1
Family *Incertae sedis* XV								
*Aminobacterium*	0	0	0	0	1	0	0	0
U_ *Incertae sedis* XV	0	0	0	0	1	0	0	0
Family *Lachnospiraceae*								
*Butyrivibrio*	4	1	2	2	6	2	3	3
*Syntrophococcus*	1	1	1	1	1	2	1	1
*Roseburia*	1	1	1	1	1	1	1	1
*Lachnobacterium*	1	1	1	1	1	1	1	1
*Lachnospiraceae incertae sedis*	6	6	6	6	7	8	7	8
*Pseudobutyrivibrio*	1	0	1	1	1	1	1	1
U_*Lachnospiraceae*	8	8	6	12	13	13	10	17
Order U*_Clostridiales*	2	1	2	7	4	4	4	10
Class U*_Clostridia*	1	1	1	0	3	1	1	1
***Class Erysipelotrichi***	**1**	**1**	**1**	**2**	**3**	**3**	**2**	**2**
*Bulleidia*	1	1	1	2	2	1	2	2
U_*Erysipelotrichaceae*	0	0	0	0	0	1	0	0
U_*Firmicutes*	0	0	0	0	1	1	0	0
**Phylum *Bacteroidetes***	**31**	**42**	**28**	**49**	**50**	**53**	**47**	**64**
***Class Bacteroidia***	**31**	**42**	**28**	**48**	**49**	**52**	**47**	**62**
*Porphyromonadaceae*								
*Dysgonomonas*	0	0	0	0	0	0	1	0
*Paludibacter*	0	0	0	1	0	1	1	1
*Parabacteroides*	0	0	0	0	0	0	0	1
U_*Porphyromonadaceae*	1	1	1	2	1	2	1	3
*Prevotellaceae*								
*Hallella*	0	1	0	1	1	1	1	1
*Prevotella*	16	24	18	27	27	31	31	37
U_*Prevotellaceae*	6	8	3	6	9	8	4	6
U_*Bacteroidales*	8	8	6	11	11	9	8	13
U_*Bacteroidetes*	0	0	0	1	1	1	0	2
**Phylum *Actinobacteria***	**1**	**0**	**0**	**0**	**1**	**0**	**0**	**1**
***Class Actinobacteria***	**1**	**0**	**0**	**0**	**1**	**0**	**0**	**1**
*Actinomyces*	1	0	0	0	1	0	0	0
*Cellulomonas*	0	0	0	0	0	0	0	1
**Phylum *Fibrobacteres***	**0**	**0**	**0**	**0**	**1**	**1**	**0**	**0**
*Fibrobacter*	0	0	0	0	1	1	0	0
**Phylum *Proteobacteria***	**0**	**1**	**1**	**0**	**4**	**2**	**2**	**2**
*Aquabacterium*	0	0	0	0	0	0	0	1
*Desulfobulbus*	0	0	0	0	0	0	0	1
*Succinivibrio*	0	0	0	0	2	1	1	0
*Citrobacter*	0	1	1	0	1	1	1	0
U_*Proteobacteria*	0	0	0	0	1	0	0	0
**Phylum *Spirochaetes***	**0**	**1**	**0**	**1**	**0**	**2**	**0**	**2**
*Treponema*	0	1	0	1	0	2	0	2
**Phylum *Tenericutes***	**0**	**0**	**0**	**0**	**1**	**0**	**0**	**1**
Anaeroplasma	0	0	0	0	1	0	0	1
**Phylum *Verrucomicrobia***	**0**	**0**	**0**	**1**	**0**	**0**	**0**	**1**
Subdivision 5 *Incertae sedis*	0	0	0	1	0	0	0	1
**Phylum *TM7***	**1**	**1**	**1**	**1**	**1**	**1**	**1**	**1**
*TM7 genera incertae sedis*	1	1	1	1	1	1	1	1
Total	80	83	69	109	135	121	108	153

*U, unclassified; C, control (without any essential oil); GAO, garlic oil; PEO, peppermint oil; ORO, origanum oil; IS, incertae sedis*.

### Effects of EOs on populations of ruminal bacteria

The effect of the tested EOs was assessed on individual bacterial populations as reflected by changes in their relative abundance. Overall, 67 individual OTUs showed significant differences (*P* ≤ 0.05) and 44 OTUs tended (0.05 <P ≤ 0.10) to change in relative abundance in response to the EOs treatments. The populations in the following phyla were examined closely as they represent most of the OTUs detected:

#### Phylum *firmicutes*

In the phylum *Firmicutes*, 42 individual OTUs showed significant (*P* ≤ 0.05) differences (Table 2) and 27 OTUs tended (0.05 < *P* ≤ 0.10) to differ (Table S1) in relative abundance among the EO treatments (Table 2 and Table S1). Compared with the control, the following OTUs were decreased significantly by ORO (Table 2): *Succiniclasticum*_9 (a taxon name followed by “_” and a number represents a specific species-equivalent OTU within that taxon), unclassified *Ruminococcaceae*_20, unclassified *Ruminococcaceae*_49, *Syntrophococcus*_1, *Roseburia*_1, *Lachnospiraceae incertae sedis*_69, and unclassified *Lachnospiraceae*_137. On the other hand, other OTUs in this phylum, including unclassified *Ruminococcaceae*_132, and unclassified *Clostridiales*_73 were increased in response to the ORO addition. The relative abundance of *Lachnospiraceae incertae sedis*_69 was lower for the GAO treatment than for the control. However, GAO increased the relative abundance of unclassified *Ruminococcaceae*_132, unclassified *Ruminococcaceae*_75, *Clostridium*_2, *Clostridium*_9, and unclassified *Lachnospiraceae*_49. For PEO, some of the OTUs related to *Syntrophococcus sucromutans*, *Succiniclasticum ruminis*, together with some OTUs unclassified within the families *Ruminococcaceae_*49 and *Lachnospiraceae_*69 were decreased, whereas other OTUs classified to *Roseburia, Prevotella, Pseudobutyrivibrio*, and unclassified within *Ruminococcaceae, Lachnospiraceae, Clostridiales, Bacteroidales* and Ad-C-8H were increased notably.

**Table 2:** **Effects of essential oils on populations of the ruminal bacteria in the phylum *Firmicutes* [only the OTUs with significant (*P* ≤ 0.05) changes in relative abundance are shown]**.

**Bacterial OTU**	**RDP ID**	**CTRL**	**ORO**	**GAO**	**PEO**	**SEM**	***P*-value**
*Streptococcus*_18	S001093592	0.56a	1.05a	0.87a	3.21b	0.224	<0.001
*Succiniclasticum*_9	S000566516	1.28b	0.61a	1.31b	1.47b	0.094	0.001
*Mitsuokella*_17	S000891012	0.71a	0.68a	0.56a	3.39b	0.278	<0.001
*Papillibacter*_10	S000823615	0.48a	0.51a	0.40a	2.59b	0.299	0.002
*Papillibacter*_13	S001146016	1.05a	1.33a	0.97a	2.15b	0.115	<0.001
*Ruminococcus*_34	S000991018	1.27a	1.19a	0.92a	2.61b	0.182	0.001
R*uminococcus*_59	S001144527	0.67a	0.73a	0.63a	1.55b	0.100	<0.001
U_*Ruminococcaceae*_120	S000990889	0.00a	0.00a	0.08a	0.62b	0.132	0.028
U_*Ruminococcaceae*_132	S000991199	2.92a	6.80b	6.47b	8.21b	1.119	0.050
U_*Ruminococcaceae*_149	S001159924	0.36ab	0.49b	0.24a	1.37c	0.050	<0.001
U_*Ruminococcaceae*_168	S001144293	56.68a	58.89a	59.67a	98.93b	5.266	0.001
U_*Ruminococcaceae*_20	S000560533	0.75bc	0.13a	0.21ab	0.83c	0.179	0.050
U_*Ruminococcaceae*_49	S000616063	23.11c	9.47ab	21.48c	3.77a	3.733	0.025
U_*Ruminococcaceae*_72	S000650474	0.02a	0.01a	0.15a	2.80b	0.056	<0.001
*Clostridium*_2	S000016649	0.38a	0.27a	0.62b	1.43c	0.070	<0.001
U_*Peptococcaceae*	S001382058	0.28a	0.28a	0.13a	5.69b	0.202	<0.001
*Butyrivibrio*_58	S000438451	4.54ab	3.04a	5.48b	6.97c	0.754	0.034
*Syntrophococcus*_1	S000389024	79.98b	46.12a	67.50b	49.46a	4.734	0.003
*Roseburia*_1	S000561181	7.40b	4.10a	5.35ab	16.22c	0.822	<0.001
LIS_52	S000926226	0.93a	0.69a	0.89a	1.61b	0.107	0.002
LIS_61	S001148837	0.21a	0.10a	0.08a	0.89b	0.053	<0.001
LIS_69	S000823633	5.10b	3.20a	3.43a	5.26b	0.432	0.017
LIS_80	S000980403	19.15a	12.46a	21.68a	40.59b	4.227	0.008
*Pseudobutyrivibrio*_8	S000126942	1.38a	0.57a	1.16a	5.76b	0.616	0.001
U_*Lachnospiraceae*_111	S000806419	0.02a	0.00a	0.20a	1.30b	0.079	<0.001
U_*Lachnospiraceae*_137	S000903858	2.45b	0.94a	2.06b	0.67a	0.245	0.002
U_*Lachnospiraceae*_156	S001144589	0.28a	0.21a	0.05a	2.35b	0.131	<0.001
U_*Lachnospiraceae*_198	S001144193	0.06a	0.00a	0.11a	7.42b	0.120	<0.001
U_*Lachnospiraceae*_207	S001144458	0.01a	0.03a	0.00a	0.86b	0.125	0.003
U_*Lachnospiraceae*_38	S000361672	0.95a	0.57a	0.82a	2.24b	0.255	0.007
U_*Lachnospiraceae*_51	S000566524	0.23a	0.18a	0.04a	2.20b	0.294	0.002
U_*Lachnospiraceae*_77	S000650410	16.21a	15.33a	20.82a	53.82b	6.130	0.006
U_*Lachnospiraceae*_96	S000821965	0.27a	0.11a	0.29a	1.86b	0.047	<0.001
U_*Clostridiales*_16	S000361544	0.72a	0.62a	0.64a	2.12b	0.302	0.020
U_*Clostridiales*_26	S000566650	0.27a	0.33a	0.53a	1.24b	0.132	0.028
U_*Clostridiales*_45	S000650472	1.86a	1.70a	2.37a	18.07b	2.353	0.003
U_*Clostridiales*_46	S000653837	0.16a	0.18a	0.07a	0.92b	0.101	0.001
U_*Clostridiales*_6	S000335878	0.01a	0.00a	0.00a	1.22b	0.005	<0.001
U_*Clostridiales*_73	S000888009	0.15a	0.56b	0.36ab	0.78c	0.069	0.001
U_*Clostridiales*_85	S000991126	1.80a	0.91a	1.49a	17.18b	0.290	<0.001
U_*Clostridia_*18	S000566535	0.06a	0.02a	0.04a	0.88b	0.039	<0.001
Ad-C-8H	S002495906	3.80a	5.07a	3.49a	16.21b	2.471	0.019

*U, unclassified; C, control (without any essential oil); GAO, garlic oil; PEO, peppermint oil; ORO, origanum oil; LIS, Lachnospiraceae incertae sedis*.

*Means followed by different letters in a row differ significantly (*P* ≤ 0.05) among the treatments*.

#### Phylum *bacteroidetes*

Twenty-four OTUs in the phylum *Bacteroidetes* showed a significant difference (Table 3) and 15 OTUs tended (0.05 < *P* ≤ 0.10) to differ (Table S2) in relative abundance between the EO treatments and the control, with 25 of them being members of the family *Prevotellaceae* (Table 3). All the OTUs affected by ORO showed increases in relative abundances compared with the control. These OTUs included *Prevotella*_18, _142, and_143 unclassified *Prevotellaceae*_34, and unclassified *Bacteroidales*_55, and _63. The GAO treatment did not significantly increase the relative abundance of any OTUs. Numerous OTUs had increased relative abundance in response to the addition of PEO, including OTUs in the families *Porphyromonadaceae* (*Parabacteroides*_1, unclassified *Porphyromonadaceae*_16, and _33) and *Prevotellaceae* (*Hallella*_14, *Prevotella*_2, _26, _68, _74, _115, _178, and _195, and unclassified *Prevotellaceae*_31), and OTUs remain unclassified in the order *Bacteroidales* (unclassified_*Bacteroidales*_18, _25, _55, _61, _75, _76, _170 and _217).

**Table 3:** **Effects of essential oils on populations of ruminal bacteria in the phylum *Bacteroidetes* and *Spirochaetes* [only the OTUs with significant (*P* ≤ 0.05) changes in relative abundance are shown]**.

**Bacterial OTU**	**RDP ID**	**C**	**ORO**	**GAO**	**PEO**	**SEM**	***P*-value**
*Parabacteroides*_1	S001542581	0.07a	0.00a	0.11a	0.82b	0.166	0.026
U_*Porphyromonadaceae*_16	S000704965	0.44a	0.55a	0.28a	2.78b	0.089	<0.001
U_*Porphyromonadaceae*_33	S001144889	0.10a	0.02a	0.01a	1.24b	0.162	0.002
*Prevotella*_115	S000336511	0.33a	0.57a	0.34a	1.12b	0.125	0.007
*Prevotella*_143	S001148780	0.41a	1.63b	0.12a	0.06a	0.360	0.048
*Prevotella*_178	S000959442	0.51a	0.85a	0.66a	1.79b	0.278	0.045
*Prevotella*_195	S000566509	3.00a	7.03ab	3.75a	14.57b	2.504	0.040
*Prevotella*_2	S000991225	0.18a	0.22a	0.13a	1.00b	0.044	<0.001
*Prevotella*_26	S000336499	0.05a	0.14a	0.13a	0.93b	0.178	0.025
*Prevotella*_68	S000823675	0.23a	0.69a	0.50a	3.12b	0.329	0.001
*Prevotella*_74	S000821891	1.90a	2.68a	3.25a	13.00b	1.796	0.007
U_*Prevotellaceae*_24	S000407047	0.32a	1.31b	0.33a	0.27a	0.252	0.050
U_*Prevotellaceae*_31	S000508055	0.47ab	1.25bc	0.16a	1.49c	0.254	0.018
U_*Prevotellaceae*_34	S000508062	0.40ab	2.50c	0.20a	1.28bc	0.499	0.041
U_*Bacteroidales*_170	S000991291	1.45a	2.55ab	1.06a	3.78b	0.528	0.026
U_*Bacteroidales*_26	S000361598	0.09a	0.08a	0.21a	5.74b	0.086	<0.001
U_*Bacteroidales*_29	S000361651	17.04a	34.97b	14.70a	19.49a	4.576	0.050
U_*Bacteroidales*_55	S000566697	0.26a	0.58b	0.14a	2.22c	0.091	<0.001
U_*Bacteroidales*_61	S001143822	0.55a	1.25ab	0.76a	2.02b	0.323	0.050
U_*Bacteroidales*_63	S000566794	1.24a	6.59b	0.65a	0.63a	1.286	0.030
U_*Bacteroidales*_75	S000650445	0.07a	0.00a	0.00a	2.25b	0.083	<0.001
U_*Bacteroidales*_76	S000650383	0.25a	0.37a	0.18a	1.22b	0.097	<0.001
U_*Bacteroidetes*_18	S000361599	0.21a	0.46a	0.14a	1.93b	0.099	<0.001
U_*Bacteroidetes*_25	S000404396	0.07a	0.06a	0.05a	0.95b	0.056	<0.001

*U, unclassified; C, control (without any essential oil); GAO, garlic oil; PEO, peppermint oil; ORO, origanum oil*.

*Means followed by different letters in a row differ significantly (P ≤ 0.05) among the treatments*.

#### Phylum *spirochaetes*

Only three OTUs in the phylum *Spirochaetes* showed a tendency to change in relative abundance in response to the EO treatments (Table S2).

### Overall effect on bacterial communities

**Figure 2:**
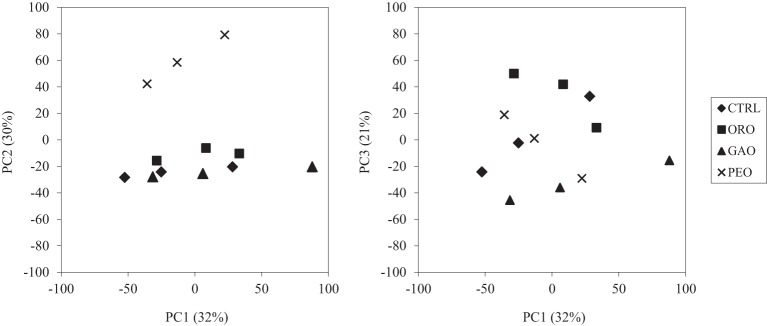
**PCA plots of the bacterial OTUs detected by the RumenBactArray among the *in vitro* ruminal cultures**. CTRL, control; ORO, origanum oil; GAO, garlic oil; PEO, peppermint oil. All the treatments were in triplicates.

Much of the variance (83%) was explained by the first three principal components (Figure 2). As shown along the PC2 axis, the PEO treatment resulted in a more distinct ruminal bacterial community than the control and the other EO treatments. The other two EOs, however, did not result in distinct bacterial communities compared to the control. Variations among the three replicates of each treatment were primarily displayed along the PC1 axis. The MANOVA also showed that the bacterial community was not significantly affected by ORO (*P* = 0.17) or GAO (*P* = 0.15) when compared to that of the control, but was significantly affected by PEO (*P* = 0.001). Among the three EO treatments, the ORO and the GAO treatments did not have significantly different (*P* = 0.089) bacterial community, but the PEO treatment had different bacterial community than the ORO (*P* = 0.036) and the GAO (*P* < 0.001) treatments (data not shown).

### Correlation between OTUs and rumen fermentation characteristics

**Figure 3:**
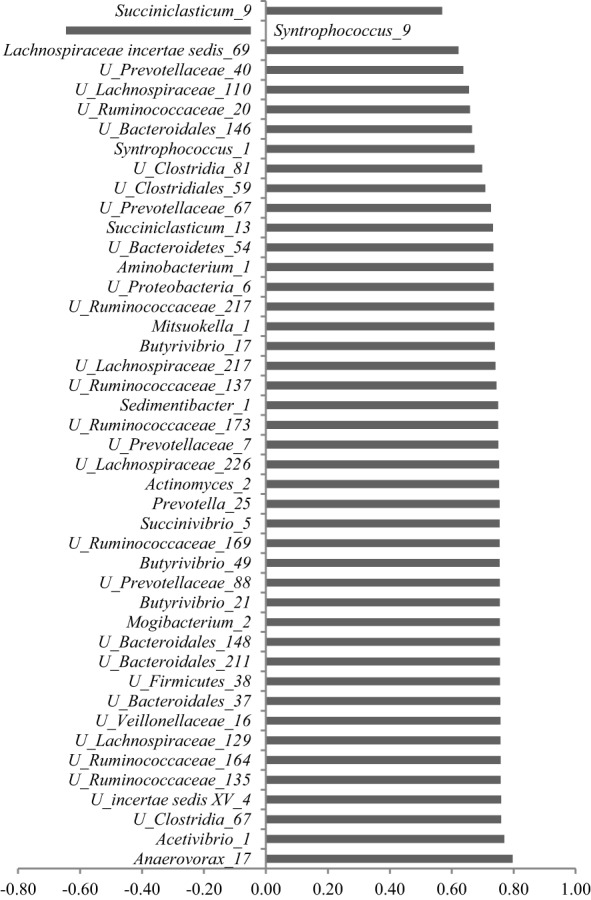
**Significant (*P* ≤ 0.05) Pearson correlation coefficients (*r*) between methane production and the relative abundances of OTUs**.

As determined by Pearson correlation coefficients (Table 4), 36 OTUs were negatively and 9 OTUs were positively correlated with DMD; 17 OTUs were negatively and 6 OTUs were positively correlated with ammonia concentrations; 3 OTUs were negatively and 29 OTUs were positively correlated with acetate concentrations; 39 OTUs were negatively and 5 were positively correlated with propionate concentration; and 5 OTUs were positively and 37 were positively correlated with butyrate concentrations. Methane production correlated positively with 43 OTUs and negatively with only one OTU, i.e., *Syntrophococcus*_9 (Figure 3). Only four OTUs (i.e., *Syntrophococcus*_1, *Syntrophococcus*_9, *Succiniclasticum*_9, and *Lachnospiraceae incertae sedis*_69) that correlated with methane production were associated with VFA profile and DM digestion.

**Table 4:** **Significant (*P* ≤ 0.05) Pearson correlation coefficients (*r*) between relative abundances of operational taxonomic units (OTUs) and some of the rumen fermentation characteristics**.

**OTUs**	**DMD**		**Ammonia**		**Acetate**		**Propionate**		**Butyrate**	
	***r***	***p*-value**	***r***	***p*-value**	***r***	***p*-value**	***r***	***p*-value**	***r***	***p*-value**
U_*Prevotellaceae*_34	−0.86	<0.001	−0.68	0.012	0.67	0.014	−0.79	0.002	0.71	0.008
U_*Prevotellaceae*_57	−0.83	<0.001	−0.70	0.01	0.73	0.006	−0.82	0.001	0.72	0.006
*Prevotella*_210	−0.81	0.001	−0.65	0.018	0.69	0.010	−0.81	0.001	0.73	0.005
U_*Prevotellaceae*_41	−0.80	0.001	−0.61	0.03	0.65	0.020	−0.77	0.002	0.71	0.008
*Hallella*_14	−0.80	0.001	−0.59	0.039	0.54	0.064	−0.69	0.011	0.65	0.02
*Sporobacter*_24	−0.80	0.001	−0.68	0.012	0.64	0.021	−0.74	0.005	0.66	0.017
*Bulleidia*_8	−0.80	0.001	−0.65	0.019	0.71	0.008	−0.78	0.002	0.68	0.013
U_*Veillonellaceae*_10	−0.80	0.001	−0.60	0.037	0.72	0.007	−0.76	0.003	0.63	0.026
*Ruminococcus*_26	−0.79	0.001	−0.60	0.035	0.69	0.010	−0.81	0.001	0.73	0.006
U_*Bacteroidales*_63	−0.79	0.001	−0.60	0.037	0.76	0.003	−0.84	<0.001	0.73	0.005
*Treponema*_8	−0.79	0.001	−0.65	0.02	0.75	0.004	−0.82	0.001	0.71	0.007
U_*Firmicutes*_2	−0.79	0.002	−0.65	0.02	0.74	0.004	−0.83	0.001	0.73	0.006
*Mitsuokella*_14	−0.78	0.002	−0.60	0.036	0.75	0.004	−0.80	0.001	0.68	0.012
*Syntrophococcus*_9	−0.78	0.002	−0.65	0.02	0.67	0.015	−0.80	0.001	0.74	0.005
*Succiniclasticum*_9	0.77	0.002	0.63	0.026	−0.72	0.007	0.85	<0.001	−0.77	0.003
*Syntrophococcus*_1	0.77	0.003	0.70	0.009	−0.41	0.187	0.63	0.025	−0.70	0.009
*Sporobacter*_27	−0.77	0.003	−0.62	0.027	0.63	0.026	−0.74	0.004	0.69	0.011
U_*Lachnospiraceae*_157	−0.76	0.003	−0.64	0.021	0.73	0.006	−0.81	0.001	0.71	0.009
LIS_95	−0.76	0.003	−0.64	0.023	0.72	0.006	−0.80	0.001	0.70	0.01
*Prevotella*_127	−0.76	0.003	−0.50	0.091	0.52	0.078	−0.71	0.008	0.69	0.011
U_*Prevotellaceae*_24	−0.76	0.003	−0.56	0.055	0.68	0.013	−0.80	0.001	0.73	0.005
*Prevotella*_91	−0.74	0.004	−0.55	0.059	0.43	0.160	−0.66	0.016	0.70	0.009
*Prevotella*_143	−0.74	0.005	−0.54	0.067	0.77	0.003	−0.81	0.001	0.67	0.015
U_*Lachnospiraceae*_124	−0.73	0.006	−0.51	0.085	0.42	0.173	−0.66	0.018	0.69	0.01
*Prevotella*_108	−0.73	0.006	−0.53	0.071	0.46	0.127	−0.64	0.022	0.64	0.021
U_*Bacteroidales*_29	−0.71	0.007	−0.47	0.12	0.59	0.040	−0.75	0.003	0.69	0.011
*Prevotella*_18	−0.71	0.008	−0.45	0.136	0.60	0.035	−0.73	0.006	0.67	0.015
*Prevotella*_122	−0.70	0.01	−0.46	0.13	0.33	0.290	−0.57	0.048	0.62	0.029
U_IS_XIII_4	−0.69	0.01	−0.49	0.104	0.70	0.010	−0.74	0.005	0.62	0.029
U_*Prevotellaceae*_47	−0.69	0.011	−0.50	0.096	0.75	0.004	−0.74	0.004	0.59	0.041
U_*Prevotellaceae*_31	−0.66	0.017	−0.48	0.111	0.28	0.384	−0.42	0.171	0.42	0.174
U_*Lachnospiraceae*_137	0.66	0.017	0.53	0.074	−0.19	0.559	0.45	0.136	−0.55	0.057
U_*Erysipelotrichaceae*_10	−0.66	0.018	−0.44	0.151	0.70	0.010	−0.73	0.006	0.61	0.033
U_*Ruminococcaceae*_23	−0.65	0.019	−0.54	0.068	0.47	0.118	−0.63	0.024	0.64	0.021
*Prevotella*_238	−0.64	0.021	−0.39	0.203	0.44	0.144	−0.63	0.025	0.63	0.026
*Butyrivibrio*_25	0.63	0.023	0.39	0.204	−0.40	0.191	0.47	0.114	−0.39	0.208
*Prevotella*_142	−0.63	0.024	−0.45	0.138	0.58	0.044	−0.72	0.007	0.68	0.013
LIS_32	0.61	0.033	0.38	0.222	−0.39	0.208	0.54	0.064	−0.56	0.051
U_*Lachnospiraceae*_26	−0.60	0.037	−0.41	0.178	0.45	0.134	−0.66	0.017	0.66	0.018
*Prevotella*_252	−0.60	0.037	−0.41	0.187	0.46	0.130	−0.66	0.018	0.65	0.02
*Treponema*_3	−0.59	0.038	−0.41	0.186	0.45	0.134	−0.66	0.018	0.65	0.019
LIS_76	0.58	0.046	0.36	0.249	−0.29	0.356	0.50	0.096	−0.57	0.05
*Dialister*_1	0.57	0.047	0.38	0.22	−0.20	0.533	0.43	0.154	−0.52	0.076
U_*Ruminococcaceae*_49	0.57	0.048	0.42	0.169	−0.19	0.556	0.36	0.248	−0.41	0.18
LIS_37	0.57	0.049	0.37	0.232	−0.50	0.094	0.60	0.034	−0.55	0.059
U_*Clostridia*_81	0.42	0.175	0.58	0.045	−0.08	0.798	0.33	0.284	−0.47	0.114
U_*Clostridiales*_59	0.42	0.175	0.58	0.046	−0.12	0.721	0.36	0.251	−0.49	0.104
U_*Lachnospiraceae*_110	0.42	0.167	0.58	0.046	−0.38	0.223	0.49	0.1	−0.50	0.096
*Mitsuokella*_1	0.39	0.21	0.56	0.054	−0.06	0.858	0.33	0.295	−0.49	0.103
*Butyrivibrio*_58	0.47	0.118	0.33	0.288	−0.82	0.001	0.69	0.011	−0.44	0.143
U_*Lachnospiraceae*_46	−0.52	0.078	−0.29	0.361	0.70	0.010	−0.64	0.021	0.47	0.121
U_*Ruminococcaceae*_214	−0.55	0.061	−0.31	0.325	0.66	0.018	−0.57	0.048	0.38	0.22
LIS_80	0.25	0.434	0.14	0.672	−0.66	0.018	0.53	0.069	−0.34	0.273
U_*Bacteroidales*_207	−0.43	0.158	−0.31	0.321	0.63	0.026	−0.40	0.197	0.10	0.754
U_*Lachnospiraceae*_204	−0.38	0.222	−0.34	0.274	0.62	0.027	−0.49	0.102	0.24	0.453
U_*Lachnospiraceae*_172	−0.47	0.114	−0.19	0.551	0.59	0.041	−0.55	0.062	0.39	0.206
*Bulleidia*_7	0.48	0.109	0.25	0.431	−0.51	0.088	0.60	0.034	−0.55	0.059
*Prevotella*_43	−0.46	0.128	−0.21	0.515	0.41	0.176	−0.58	0.045	0.57	0.05
LIS_69	0.35	0.255	0.32	0.307	−0.37	0.236	0.52	0.076	−0.61	0.032
*Prevotella*_121	−0.54	0.066	−0.35	0.264	0.24	0.443	−0.49	0.101	0.57	0.05

*U, unclassified; LIS, Lachnospiraceae incertae sedis*.

## Discussion

The rumen bacterial community is extremely diverse, especially at the species and genus levels, collectively containing over 5200 OTUs at species level and 3500 OTUs at genus levels (Kim et al., 2011; Creevey et al., 2014). In most studies that examined the effects of dietary interventions, including mitigation of methane emission by anti-methanogenic compounds or substances, only a few small groups of bacteria were analyzed (Patra and Yu, 2012; Fiorentini et al., 2013; Martínez-Fernández et al., 2014). The narrow scopes of these studies prevent full revelation of the actual impact of the feed additives on the rumen bacterial communities and the effect on the interactions among different bacteria. As a result, it is often difficult to understand the mode(s) of action or explain the observed efficacy of the feed additives. The rumen bacterial populations are highly dynamic in how they respond to changes in diet, feed additives, feeding regiments, and physiological status of the ruminants (Wang et al., 2009; Li et al., 2012; Wu et al., 2012). Most of these effectors typically cause fluctuations of bacterial populations, rather than completely eliminate bacterial populations or bring about the emergence of new bacterial populations. In the present study, RumenBactArray was used to comparatively examine the impact of EOs on rumen bacteria in a semi-quantitative manner.

The EOs evaluated affected the rumen bacterial community composition differently at species level depending upon the EO types. A number of mechanisms of action have been proposed to explain the antimicrobial properties of EOs, with chemical structures and physical properties being most important to determine their antimicrobial potency (Dorman and Deans, 2000; Burt, 2004). The presence of phenolic structure and the position of a hydroxyl group in the phenolic structure of EOs (e.g., EOs containing thymol or eugenol) can influence the antimicrobial potency of EOs (Dorman and Deans, 2000; Ultee et al., 2002). The greater antibacterial potency of ORO (containing a phenol) than PEO (containing a cyclohexane) shown in this study (Table 2) corroborates the importance of the phenolic ring to the antimicrobial activities of EOs (Ultee et al., 2002). Gram-negative bacteria are usually thought to be less susceptible to EOs than Gram-positive bacteria due to the presence of a protecting outer membrane (Dorman and Deans, 2000; Burt, 2004). Members of *Prevotella*, a Gram-negative genus, increased their populations in response to the addition of ORO and PEO, while GAO did not affect the populations of *Prevotella*. On the other hand, members of the *Firmicutes*, a largely Gram-positive phylum, were decreased by all the three EOs, and bacterial groups in the class *Clostridia*, which contains most of the Gram-positive rumen bacteria, were decreased by ORO and GAO, but not by PEO. Evidently, effects of EOs on rumen bacteria are both species and EO type dependent.

Addition of a few EOs to diets fed to ruminants has been shown to decrease degradation of starch and protein, improve rumen fermentation, and inhibit methanogenesis and biohydrogenation of polyunsaturated fatty acids in the rumen (McIntosh et al., 2003; Calsamiglia et al., 2007; Patra, 2011), which was considered to be a consequence of modification of microbial populations in the rumen. The three EOs evaluated in this study decreased the number of members in the genus *Butyrivibrio*. *Butyrivibrio fibrisolvens*, a major cultured species of butyrate-producing Gram-positive bacteria ubiquitous in the rumen, was found to be very sensitive to a blend of EOs (McIntosh et al., 2003). The population of this species was inhibited by ORO, but not by PEO at the similar dose level (Patra and Yu, 2014). Zhu et al. (2014) also reported decreased 16S rRNA gene clones related to *Butyrivibrio proteoclasticus* and *Pseudobutyribrio ruminis* in the rumen of goats fed 0.8 g/d of GAO. In the present study, the ORO supplementation decreased the relative abundances of many OTUs of *S. sucromutans*, *Lachnospiraceae incertae sedis*, and unclassified *Ruminococcaceae*, but increased that of *S. ruminis*, and some members of *Prevotella*, and unclassified *Bacteroidales*, *Lachnospiraceae*, and *Prevotellaceae*. *Butyrivibrio*, *Anaerovoax* (member of the *Lachnospiraceae incertae sedis* family), and unclassified *Clostridiales* and *Ruminococcaceae* have been suggested to have a key role in rumen biohydrogenation (Huws et al., 2011). Thus, modification of these microbial compositions by EOs may be associated with changes in the rumen biohydrogenation process (Lourenço et al., 2008; Ramos-Morales et al., 2013) and the increased concentrations of conjugated linoleic acid (CLA) and other poly unsaturated fatty acids in milk and tissues of ruminants fed EOs (Morsy et al., 2012; Mandal et al., 2014).

Supplementation of ORO and PEO increased while GAO did not affect the predominant representatives of *Prevotella* in the present study. The relative abundances of several *Prevotella* OTUs increased in response to EOs probably due to reduced competition from other bacteria that were inhibited by EOs. The genus *Prevotella* genus is present in the rumen across a variety of diets and exhibits substantial metabolic diversity (Petri et al., 2013). This genus comprises species that appear to be involved in protein degradation in the rumen (Wallace et al., 1997). It was suggested that EOs can decrease protein degradation and ammonia concentrations in the rumen (McIntosh et al., 2003; Patra, 2011). Indeed, addition of ORO significantly lowered ammonia concentrations, while PEO numerically reduced ammonia concentration at the dose level of 0.50 g/L in the mixed *in vitro* rumen cultures (Patra and Yu, 2014). The reduced ammonia production was also associated with reduced abundances of the major protein-degrading and amino acid-fermenting bacteria (Patra and Yu, 2014). Thus, it appears that few members of *Prevotella* or other OTUs which were not detected in this study may play a major role in protein metabolism in the rumen.

Inclusion of PEO increased relative abundances of several unclassified bacterial species of *Ruminococcaceae* and *Lachnospiraceae*. Zhu et al. (2014) also noted that some bacterial 16S rRNA genes classified to the genus *Ruminococcus* and other genera within the family *Ruminococcaceae* were specifically found in the GAO group. The number of OTUs of the unclassified *Ruminococcaceae* was lower in all the EO treatments than in the control. In our earlier study (Patra and Yu, 2012), ORO and PEO decreased the abundance of *R. flavefaciens* and *R*. *albus*. The members of *Ruminococcaceae* contribute significantly to fiber metabolism (Koike and Kobayashi, 2009), and thus EOs at high doses may decrease fiber digestion (Patra and Yu, 2012). In a previous study, DGGE-based analysis has shown that PEO and ORO resulted in bacterial communities that were distinctly different from that of control at the dose selected in this study (Patra and Yu, 2012). However, in the present study, the bacterial communities were only different between the PEO treatment and the control. This discrepancy was likely due to techniques used for community analysis. Much fewer bacterial groups can be detected by DGGE-based analysis than by microarray.

Addition of EOs changed the abundances of a few less-known bacteria. The relative abundance of *Syntrophococcus* was considerably decreased by ORO and PEO. The role of *S*. s*ucromutans* in the rumen metabolism is not well-understood, but it produces acetate only from pyruvate and various carbohydrates (Krumholtz and Bryant, 1986). All three EOs also lowered the abundances of the genus *Succinivibrio*. The representatives of this genus increased when ruminants were fed high levels of grains or rapidly degradable carbohydrate such as diets rich in starch (Patterson and Hespell, 1985; O'Herrin and Kenealy, 1993). *Succinivibrio dextrinosolvens* may be one of the major rumen bacteria fermenting dextrin and levans in starch-based diets (Patterson and Hespell, 1985). Because all the three EOs were inhibitory to *Succinivibrio*, the effect of EOs on starch metabolism in the rumen may be attributed to their effect on this group of bacteria. The relative abundance of *Acetanaerobacterium*, a genus of Gram-positive bacteria, was generally lower in the EO treatments than in the control. The role of this bacterial genus in rumen metabolism is poorly understood except fermenting sugars to acetate from sugars (Chen and Dong, 2004). One OTU of the genus *Actinomyces* was detected in the control, while it was not detected in any of the EO treatments. *Actinomyces ruminicola* hydrolyzes xylan and starch and ferment several kinds of mono-, di-, and oligosaccharides (An et al., 2006). Much of the basic understandings on the rumen microbiome and the specific roles of microorganisms in rumen metabolism were obtained from studies on individual bacteria isolated in pure cultures (Petri et al., 2013). However, the majority of the ruminal microorganisms remain uncultured (Kim et al., 2011; Creevey et al., 2014), and thus their specific roles in the overall rumen fermentation remain to be elucidated. A large amount of the rumen bacteria will remain to be uncultured in the foreseeable future. As shown in the present study, the RumenBactArray can help in establishing associations between bacterial abundance and fermentation variables in the rumen in repeated studies.

Feed degradability and VFA profiles in the rumen are some of the most important parameters indicative of bacterial metabolism. Thus, correlation coefficients were determined to investigate association between OTUs and major rumen fermentation characteristics. Overall 60 OTUs were associated with the changes in DMD, ammonia concentration, and VFA profiles, which suggest that the dynamic changes of these OTUs in response to the EO treatments are probably responsible for or caused by the shifts in these fermentation characteristics. Correlation between specific bacteria and VFA profiles in the rumen has been scarcely reported, with a positive correlation being noted between abundance of genus *Butyrivibrio* and proportion of butyrate (Mohammed et al., 2014), but significant correlations were noted between VFA concentrations and several bacterial community in the feces of cattle (Mao et al., 2012). Future studies using quantitative tools, such as the RumenBactArray, may provide opportunities to determine the bacteria that are associated with some of the important fermentation characteristics in the rumen. In this study, 17 OTUs were found to have negative correlation with DMD, concentration of ammonia, and molar percentage of propionate, but positive correlation with molar percentages of acetate and butyrate, irrespective of EO treatments. Only four OTUs that were associated with VFA characteristics correlated with methane production. As noted by Mao et al. (2012), changes in bacterial populations and VFA profile depend on many other variables such as competition among bacteria for substrates, synthesis of antimicrobial agents, and bacterial metabolism. Future studies are needed to confirm these correlations and to determine their causality. Although not confirmatory, the results of the present study also suggest that a relatively small group of rumen bacteria may be responsible for and/or indicative of each of the important characteristics of feed digestion and fermentation. The members of these groups can be identified in future studies (both *in vitro* and *in vivo*) in which each of these feed digestion and fermentation characteristics is changed intentionally through dietary interventions or by addition of those particular bacterial groups *in vitro* or *in vivo*. Conceivably, once confirmed, these responsible or indicator groups of bacteria, rather than the entire rumen microbiome, can be specifically analyzed more effectively to support nutritional studies of ruminant animals.

The *in vitro* rumen fermentation technique is useful to assess digestibility, rumen fermentation characteristics, and microbial community structure influenced by feeds and feed additives, which is easy to conduct and inexpensive compared with *in vivo* measurements. However, the extrapolation of the results of *in vitro* studies to *in vivo* conditions may be sometimes unrepresentative due to continuous absorption of VFA, neutralization by saliva, changes in the microbial ecosystem such as a decrease in total microbial biomass and shifts in bacterial community composition, and low number of fungi and protozoa *in vitro* (Soto et al., 2012). In addition, different *in vitro* conditions can have different effects on certain rumen microbial populations (Weimer et al., 2011). Besides, the number of replicate samples for statistical analyses was low, especially for a microbial ecology study. Thus, the results obtained in this *in vitro* study might have limitations, and should be interpreted accordingly.

In conclusion, this study demonstrated for the first time using microarray analysis that EOs can affect the population dynamics of a number of bacteria, especially those in the families *Prevotellaceae*, *Butyrivibrio*, *Lachnospiraceae*, and *Ruminococcaceae*, in an EO type-dependent manner. Many bacterial OTUs were found to be associated with changes in feed digestibility and rumen fermentation characteristics, which may explain the modulation of rumen fermentation due to feed additives.

### Conflict of interest statement

The authors declare that the research was conducted in the absence of any commercial or financial relationships that could be construed as a potential conflict of interest.
