# Supplementary material for: Essential oils affect populations of some rumen bacteria in vitro as revealed by microarray (RumenBactArray) analysis
Source: Front Microbiol. 2015 Apr 10;6:297. doi: 10.3389/fmicb.2015.00297 (PMC4392297; doi:10.3389/fmicb.2015.00297)
Supplement: Supplementary file 1 [file Table1.PDF]

SUPPLEMENTARY DATA for Patra and Yu

Table S1. Effects of essential oils on populations of some of the ruminal bacteria in the phylum *Firmicutes* (shown are the OTUs having tendency (0.05<P≤0.10) to change in relative abundance in response to the EO treatments).

Bacterial OTU	RDP ID	CTRL	ORO	GAO	PEO	SEM	P-value
*Bacillus*_1	S000541778	6.85b	4.38ab	3.74a	7.09b	0.987	0.097
*Succiniclasticum*_1	S000131229	24.95ab	33.56b	36.05b	14.31a	5.055	0.059
*Mitsuokella*_14	S000566768	0.40a	1.99b	0.17a	0.49a	0.471	0.089
U_*Veillonellaceae*_10	S000508235	0.20a	0.68b	0.07a	0.38ab	0.149	0.090
*Mogibacterium*_4	S000561072	0.01a	0.04a	3.83b	0.00a	1.006	0.066
*Acetanaerobacterium*_1	S000705020	2.17b	0.02a	0.00a	0.06ab	0.558	0.062
*Ruminococcus*_26	S000607272	1.37a	4.54b	1.18a	1.49a	0.828	0.060
*Sporobacter*_24	S000650448	0.09a	0.69b	0.07a	0.41ab	0.168	0.092
U_*Ruminococcaceae*_221	S001148852	0.00a	0.09a	0.00a	1.85b	0.481	0.066
U_*Ruminococcaceae*_75	S001143789	0.00a	0.01a	0.57b	0.01a	0.151	0.068
*Clostridium*_7	S000903866	0.04a	0.00a	0.00a	0.67b	0.188	0.092
*Clostridium*_9	S000927679	0.00a	0.00a	5.61b	0.16a	1.437	0.060
*Butyrivibrio*_25	S000561168	0.71ab	0.03a	1.24b	0.09a	0.304	0.068
*Syntrophococcus*_9	S001144221	0.09a	0.79b	0.18a	0.21a	0.182	0.088
LIS_12	S000335897	25.06b	10.72ab	18.07ab	6.11a	4.894	0.102
LIS_95	S001144204	0.06a	1.56b	0.02a	0.07a	0.428	0.088
U_*Lachnospiraceae*_132	S000888041	0.00a	0.00a	0.00a	1.00b	0.263	0.065
U_*Lachnospiraceae*_157	S000991116	0.02a	1.34b	0.00a	0.01a	0.367	0.079
U_*Lachnospiraceae*_26	S001146298	0.00a	1.60b	0.00a	0.00a	0.428	0.071
U_*Lachnospiraceae*_46	S000560514	0.70a	1.02b	0.45a	0.27a	0.188	0.093
U_*Lachnospiraceae*_49	S000561108	0.01a	0.14a	1.90b	0.28a	0.449	0.055
U_*Lachnospiraceae*_64	S001145543	0.29a	0.46a	0.58a	1.65b	0.328	0.069
U_*Clostridiales*_13	S000361458	0.11a	0.10a	0.12a	0.71b	0.152	0.055
U_*Clostridiales*_145	S001161820	0.13a	0.39a	0.18a	1.73b	0.389	0.060
U_*Clostridia*_11	S000561085	0.03a	0.00a	1.35b	0.02a	0.355	0.069
*Bulleidia*_7	S000990836	1.22ab	0.08a	1.22ab	2.00b	0.418	0.066
U_*Firmicutes*_2	S000335921	0.01a	0.55b	0.00a	0.01a	0.139	0.057

U, unclassified; CTRL, control (without any essential oil); GAO, garlic oil; PEO, peppermint oil; ORO, origanum oil; LIS, *LachnospiraceaeIncertae* Sedis

Means followed by different letter in a row differ significantly (P≤0.05) among the treatments.
