# Supplementary material for: Essential oils affect populations of some rumen bacteria in vitro as revealed by microarray (RumenBactArray) analysis
Source: Front Microbiol. 2015 Apr 10;6:297. doi: 10.3389/fmicb.2015.00297 (PMC4392297; doi:10.3389/fmicb.2015.00297)
Supplement: Supplementary file 2 [file Table2.PDF]

**Table S2.** Effects of essential oils on populations of some of the ruminal bacteria in the phylum *Bacteroidetes* and *Spirochaetes* (shown are the OTUs having tendency ($0.05<P\leq0.10$) to change in relative abundance in response to the EO treatments)

| Bacterial OTU | RDP ID | CTRL | ORO | GAO | PEO | SEM | P-value |
|---|---|---|---|---|---|---|---|
| **Phylum *Bacteroidetes*** | | | | | | | |
| *Hallella*_14 | S000991205 | 0.61a | 1.74b | 0.49a | 1.26b | 0.307 | 0.065 |
| *Prevotella*_127 | S001160045 | 0.81a | 2.27b | 0.72a | 1.19ab | 0.399 | 0.086 |
| *Prevotella*_142 | S000566489 | 0.86a | 3.03b | 1.31ab | 0.55a | 0.653 | 0.102 |
| *Prevotella*_145 | S000991147 | 0.01a | 0.00a | 0.03a | 4.52b | 1.207 | 0.070 |
| *Prevotella*_18 | S000823685 | 2.24a | 6.00b | 1.96a | 2.00a | 1.153 | 0.100 |
| *Prevotella*_207 | S000361403 | 0.05a | 0.14a | 8.00b | 0.54a | 2.051 | 0.066 |
| *Prevotella*_210 | S000723107 | 1.51a | 12.76b | 1.54a | 3.46a | 2.849 | 0.067 |
| *Prevotella*_226 | S000336577 | 0.06a | 0.00a | 0.00a | 4.85b | 1.278 | 0.067 |
| *Prevotella*_236 | S000361472 | 0.00a | 0.00a | 0.66b | 0.03a | 0.175 | 0.069 |
| *Prevotella*_252 | S000336544 | 0.02a | 0.97b | 0.00a | 0.03a | 0.259 | 0.076 |
| *Prevotella*_63 | S000722862 | 0.02a | 0.00a | 0.74b | 0.00a | 0.193 | 0.067 |
| *Prevotella*_70 | S000365831 | 0.50ab | 0.85b | 0.60ab | 0.16a | 0.165 | 0.092 |
| U_*Prevotellaceae*_41 | S000406925 | 1.66a | 6.11b | 1.66a | 3.05ab | 1.194 | 0.090 |
| U_*Prevotellaceae*_57 | S000977443 | 1.11a | 4.97b | 0.93a | 2.15ab | 0.979 | 0.064 |
| U_*Bacteroidales*_217 | S001277976 | 0.42a | 1.06a | 1.16a | 3.60b | 0.781 | 0.083 |
| **Phylum *Spirochaetes*** | | | | | | | |
| *Treponema*_17 | S000991276 | 0.00a | 0.07a | 0.01a | 2.15b | 0.564 | 0.068 |
| *Treponema*_3 | S000566793 | 0.02a | 1.27b | 0.00a | 0.01a | 0.341 | 0.073 |
| *Treponema*_8 | S000566578 | 0.06a | 0.80b | 0.00a | 0.06a | 0.198 | 0.061 |

U, unclassified; CTRL, control (without any essential oil); GAO, garlic oil; PEO, peppermint oil; ORO, origanum oil

Means followed by different letter in a row differ significantly ($P\leq0.05$) among the treatments.
